# The Vitamin D Decrease in Children with Obesity Is Associated with the Development of Insulin Resistance during Puberty: The PUBMEP Study

**DOI:** 10.3390/nu13124488

**Published:** 2021-12-15

**Authors:** Liliane Viana Pires, Esther M. González-Gil, Augusto Anguita-Ruiz, Gloria Bueno, Mercedes Gil-Campos, Rocío Vázquez-Cobela, Alexandra Pérez-Ferreirós, Luis A. Moreno, Ángel Gil, Rosaura Leis, Concepción M. Aguilera

**Affiliations:** 1 Department of Biochemistry and Molecular Biology II, School of Pharmacy, University of Granada, 18071 Granada, Spain; lvianapires@gmail.com (L.V.P.); esthergg@unizar.es (E.M.G.-G.); augustoanguitaruiz@gmail.com (A.A.-R.); agil@ugr.es (Á.G.); 2 Center of Biomedical Research, Institute of Nutrition and Food Technology “José Mataix”, University of Granada, Avda. del Conocimiento s/n, 18016 Armilla, Spain; 3 Nutrition Sciences Post-Graduation Program, Department of Nutrition, Federal University of Sergipe, Saint Cristopher 49100-000, Brazil; 4 Growth, Exercise, Nutrition and Development (GENUD) Research Group, University of Zaragoza, 50009 Zaragoza, Spain; gbuenoloz@yahoo.es (G.B.); lmoreno@unizar.es (L.A.M.); 5 CIBEROBN (Physiopathology of Obesity and Nutrition), Institute of Health Carlos III (ISCIII), 28029 Madrid, Spain; mercedes_gil_campos@yahoo.es (M.G.-C.); cobela.rocio@gmail.com (R.V.-C.); 6 Instituto Agroalimentario de Aragón (IA2) and Instituto de Investigación Sanitaria de Aragón (IIS Aragón), 50009 Zaragoza, Spain; 7 Department of Pediatric Endocrinology, Institute Maimónides of Biomedicine Investigation of Córdoba (IMIBIC), Reina Sofia University Clinical Hospital, University of Córdoba, Avda. Menéndez Pidal s/n, 14004 Cordoba, Spain; 8 Unit of Investigation in Nutrition, Growth and Human Development of Galicia, Pediatric Department (USC), Instituto de Investigación Sanitaria de Santiago de Compostela (IDIS), University Clinical Hospital, 15706 Santiago de Compostela, Spain; alexandra15pf@gmail.com; 9 Instituto de Investigación Biosanitaria ibs., 18012 Granada, Spain

**Keywords:** vitamin D, cardiometabolic risk factors, puberty, obesity, insulin resistance, child

## Abstract

Obesity and cardiometabolic risk have been associated with vitamin D levels even in children. The objective of the present study was to evaluate the association between insulin resistance (IR), cardiometabolic risk factors, and vitamin D in children from prepubertal to pubertal stages. A total of 76 children from the PUBMEP study, aged 4–12 years at baseline, were included. Children were evaluated in prepubertal and pubertal stages. Anthropometric measurements and selected cardiometabolic risk biomarkers, such as plasma glucose, blood lipids, insulin, adiponectin, leptin, and blood pressure, and serum 25-hydroxyvitamin D (25(OH)D) were determined. Children were categorized by obesity degree and IR status combined before and after puberty. Paired *t*-test and multivariate linear regression analyses were conducted. During puberty, the increase in triacylglycerols, insulin, and HOMA-IR and the decrease in QUICKI were significantly associated with the reduction in 25(OH)D (B = −0.274, *p* = 0.032; B = −0.219, *p* = 0.019; B = −0.250, *p* = 0.013; B = 1.574, *p* = 0.013, respectively) after adjustment by BMI-z, sex, and pubertal stage. Otherwise, prepubertal non-IR children with overweight/obesity that became IR during puberty showed a significant decrease in 25(OH)D and HDL-c, and an increase in waist circumference and triacylglycerol concentrations (*p* < 0.05 for all) over time. These results suggest that changes in IR seem to be associated with an effect on 25(OH)D levels during puberty, especially in children with overweight.

## 1. Introduction

The association of vitamin D deficiency with higher body mass index (BMI), insulin resistance (IR), and cardiometabolic risk factors in observational studies with pediatric and young populations has been reported [1,2,3]. Furthermore, previous literature has described a higher prevalence of vitamin D deficiency in children and adolescents with obesity in the pubertal stage compared to prepuberty [4,5].

Lower vitamin D serum concentrations in individuals with obesity may be due to the lower bioavailability of vitamin D as a consequence of its storage in adipose tissue and the volumetric dilution related to larger body size [6,7]. Vitamin D is metabolized in the liver to 25-hydroxy-cholecalciferol (25(OH)D) and later in the kidney to 1,25-dihydroxy-cholecalciferol (1,25(OH)_2_D) [8]. While serum levels of 25(OH)D are recognized as the best marker of vitamin D nutritional status, 1,25(OH)_2_D, also known as calcitriol, binds to vitamin D receptors (VDRs), which are present in a wide variety of tissues other than bone, including pancreatic β-cells, the adipose tissue, brain, and activated T and B lymphocytes, among others [9]. Indeed, vitamin D is associated with the mechanisms responsible for insulin secretion, favoring blood glucose control [10,11]. Moreover, several studies showed that cardiometabolic risk markers are related to lower plasma vitamin D concentrations in children [12,13].

Obesity is also associated with alterations of the lipid profile and increased concentrations of circulating inflammatory biomarkers, which are closely related to IR and, consequently, hyperglycemia [9]. Additionally, puberty is a period of growth and vitamin D, as a steroid hormone, plays an important role in the regulation of calcium levels and bone metabolism [14]. In addition, vitamin D activity occurs via the binding of its active metabolite 1,25(OH)_2_D to the VDR, which is also expressed in the reproductive organs, which could partly explain the association between vitamin D and puberty [15,16]. However, there are still few longitudinal studies assessing cardiometabolic risk and vitamin D status during puberty. A study showed that prepubertal children with central obesity and suboptimal vitamin D levels had higher pubertal IR compared with those no central obesity and optimal vitamin D levels [17]. Finally, most of the population has vitamin D deficiency, and this trend has also been found in children [18,19], which highlights the importance of longitudinal studies aiming to assess vitamin D status in critical periods of life, such as puberty, or conditions such as obesity. Thus, the present study aimed to evaluate the relationship between insulin resistance, cardiometabolic risk factors, and serum 25(OH)D concentrations from prepuberty to puberty, considering BMI status, in a sample of Spanish children.

## 2. Materials and Methods

### 2.1. Subjects and Study Design

The present work included Spanish children from the PUBMEP study, a longitudinal study based on the follow-up of a cohort of children who had previously participated in the GENOBOX study [20]. GENOBOX is a case–control, multicenter study carried out in a total of 1444 Spanish children (706 males and 738 females), aged 3 to 17 years, from 2012–2015. Detailed inclusion and exclusion criteria as well as informed consent and approval by the local ethics committees where children were recruited have been reported elsewhere [21]. A subsample of 76 children (34 girls) recruited in the Clinical University Hospital of Santiago de Compostela was selected based on the following inclusion criteria: having measured twice, at prepubertal and pubertal stages, the serum 25(OH)D, homeostasis model assessment for insulin resistance (HOMA-IR), height, and weight, for the calculation of body mass index (BMI), and pubertal status recorded. All the children were initially recruited in the prepubertal stage between 2012 and 2015 (T0) and invited for follow-up medical consultation in 2018 (T1). Among the recruited children, participants who were in the pubertal period (at least Tanner II, confirmed with sex hormones), were invited to participate in the PUBMEP study. At the prepubertal stage, the children were aged 4–12.1 years, and at the pubertal time they were aged 9.7–18.1 years (mean time between measurements: 6.2 ± 2.8 years).

To assess the influence of puberty on serum concentration of 25(OH)D and the metabolic outcomes, children were categorized according to the combination of their obesity degree by Cole et al. [22] and the presence of IR by HOMA-IR cut-off points detailed below. Thus, they were allocated into six experimental groups. Group 1 consisted of normal weight and non-IR children that remained a normal weight and non-IR over time; group 2, children with overweight (OW) or obesity (OB) and non-IR who changed to normal weight and non-IR during puberty; group 3, children with OW/OB and non-IR with no change over time; group 4, children with OW/OB and IR changing to non-IR during puberty; group 5, children with OW/OB and non-IR changing to IR; and group 6, children with OW/OB and IR with no change (Supplementary Figure S1). The samples were collected throughout the year (at the 43rd parallel north), representing the different seasons to control for seasonal effects. In addition, no children took multivitamins or, specifically, vitamin D supplements.

This study was conducted according to the guidelines set out in the Declaration of Helsinki (Edinburgh 2000 revised), and all procedures were approved by the Ethics and Research Committee of Galicia Autonomous Community (2011/198 and 2016/522). Written consent was obtained from the parents of all the children.

### 2.2. Clinical Examination and Anthropometric Measurements

Anthropometric measurements were taken with the children barefoot and in their underwear. Bodyweight (kg), height (cm), and waist circumference (WC) (cm) were measured using standardized procedures, and BMI was calculated. The BMI z-scores were calculated based on the Spanish reference standards [23], and the children were classified for obesity using the BMI age- and sex-specific cut-off points proposed by Cole et al. [22].

According to the Tanner criteria, the pubertal stage was determined following the standards for pubic hair and genitalia growth in boys and breast and pubic hair development in girls through a physical examination by pediatric endocrinologists [24]. It was confirmed by the determination of sexual hormones in serum. Blood pressure was measured three times by the same examiner following international recommendations [25].

### 2.3. Biochemical Analyses

After 10 h of overnight fasting and rest, blood samples were drawn by venipuncture. The samples were protected from sunlight and refrigerated. The serum and plasma were separated and cryopreserved at −80 °C until the analysis was conducted.

The insulin concentration was analyzed by chemiluminescent microparticle immunoassay. Serum concentrations of glucose, lipids (total cholesterol, triacylglycerols (TAG), high-density lipoprotein cholesterol (HDL-c), and low-density lipoprotein cholesterol (LDL-c)) were measured by spectrophotometry.

Plasma adipokines (adiponectin and leptin) were analyzed using XMap technology (Luminex Corporation, Austin, TX, USA) and human monoclonal antibodies (Milliplex Map Kit; Millipore, Billerica, MA, USA), as previously reported in the literature [26].

### 2.4. Homeostasis Model Assessment for Insulin Resistance Cut-Off Points

The presence of IR in children was defined according to the HOMA insulin resistance (HOMA-IR) index. The cut-off points were obtained from a previously well-described Spanish cohort composed of children and adolescents [27]. Values of HOMA-IR ≥ 2.5 were considered as an IR indicator for the prepubertal stage [27]. For the pubertal stage, the cut-off points for IR were based on the 95th HOMA-IR percentile, considering sex (HOMA-IR ≥ 3.38 in boys and HOMA-IR ≥ 3.90 in girls) [26].

### 2.5. Analysis and Diagnostic Criteria of Vitamin D Status

Serum 25(OH)D concentrations were quantified using a direct competitive chemiluminescence immunoassay by the LIAISON method. Sensitivity of the assay was 4 ng/mL (10 nmol/L) and the intraclass correlation coefficient (CV) was 5.5% at 7.2 ng/mL, 2.9% at 35 ng/mL, and 4.8% at 128 ng/mL. The interclass CV was 12.7% at 5.91 ng/mL, 6.9% at 45.9 ng/mL, and 7.9% at 62.7 ng/mL. A duplicate of each sample was analyzed.

According to the cut-off proposed by Holick et al. [28], vitamin D ‘deficiency’ was defined as serum 25(OH)D levels <20 ng/mL, ‘sufficient for bone health’ as 20–29 ng/mL, and ‘optimal’ as ≥30 ng/mL, which is associated with additional health benefits, although values of 40–60 ng/mL are preferred.

### 2.6. Statistical Analysis

A Kolmogorov–Smirnov test was used to assess the distribution of the included variables and transformation was performed when needed. Those variables that did not achieve normality were analyzed with non-parametric tests after transformation. A chi-square test was used for comparisons of categorical variables.

A paired *t*-test or Wilcoxon signed-rank test was used to determine the statistical difference of the changes between prepubertal and pubertal stages for the considered cardiometabolic risk factor variables.

Associations between the cardiometabolic factors and 25(OH)D levels were examined using linear regression analysis and Pearson correlation coefficient. Two models were created for the cross-sectional analysis in the pubertal stage: an unadjusted model and an adjusted model, including BMI z-score, sex, and the pubertal stage reached (I–V). For the longitudinal analysis, two models were also created: one unadjusted and one adjusted by BMI z-score (T1), sex, the pubertal stage reached (I–V), and by the baseline value of the cardiometabolic biomarker 25(OH)D levels (T0). Multicollinearity was checked using the variance inflation factor (VIF)/tolerance. All models included in this study presented VIFs below 2, and the tolerance measures were ≥0.7, according to a reference for VIF < 5 and tolerance >0.2.

Delta values (Δ = T1–T0) were also obtained for each continuous variable. Comparisons of the metabolic variables were performed according to vitamin D classification for the groups of prepubertal and pubertal children using ANOVA or the Kruskal–Wallis test. Bonferroni correction was applied for multiple comparisons. A *p*-value < 0.05 was considered significant. All analyses were performed using the SPSS software (version 26).

## 3. Results

The mean age of the included children was 7.8 ± 1.9 years in the prepubertal stage, 45% being girls. The characteristics of the prepubertal and pubertal children according to the vitamin D status are presented in Table 1. The adiponectin levels were lower in prepubertal children with deficiency and insufficiency of vitamin D than those with optimal levels (*p* = 0.036); however, no difference was observed in the pubertal stage (*p* = 0.582). In addition, there were mean differences in some cardiometabolic factors, with higher values of WC, systolic and diastolic blood pressure, TAG, fasting insulin, and HOMA-IR, (all, *p* < 0.01), and lower values for LDL-c (*p* = 0.026), QUICKI (*p* < 0.01), adiponectin (*p* = 0.001), and leptin levels (*p* = 0.047) in the pubertal stage in comparison with the prepubertal stage. 

Considering the deficiency of vitamin D according to obesity degree, 50% of the prepubertal children with obesity presented deficiency in vitamin D (Supplementary Table S1). On the other hand, a total of 40.8% of the pubertal children presented obesity and serum 25(OH)D levels decreased significantly from the prepubertal to the pubertal stage (Δ = −4.0 ng/mL, *p* = 0.004) (Table 1), with 64.5% of the children with obesity having vitamin D deficiency (Supplementary Table S1).

The associations between cardiometabolic variables and 25(OH)D levels in the pubertal stage are shown in Table 2. Results showed that WC (*p* = 0.021), TAG (*p* = 0.002), insulin levels (*p* = 0.001), HOMA-IR (*p* = 0.001), and concentration of leptin (*p* = 0.032) were inversely associated with 25(OH)D levels and QUICKI (*p* = 0.001), and directly associated with this vitamin in the unadjusted model. After adjusting for BMI, sex, and the pubertal stage, the associations remained for TAG, insulin levels, HOMA-IR, and QUICKI with 25(OH)D levels (B =−0.274, *p* = 0.032; B = −0.250, *p* = 0.013; B = −0.219, *p* = 0.019; B = 1.574, *p* = 0.013).

Prospective associations between cardiometabolic variables at the prepubertal stage (T0) and 25(OH)D levels in the pubertal stage (T1) are shown in Table 3. The insulin (*p* = 0.030), HOMA-IR (*p* = 0.032), and adiponectin (*p* = 0.049) levels in the prepubertal stage (T0) were inversely associated with 25(OH)D levels in the pubertal stage (T1). Additionally, the QUICKI was directly associated with 25(OH)D levels (*p* = 0.043). However, these associations were not maintained after adjusting the analysis for 25(OH)D levels in the prepubertal stage (T0), BMI z-score (T1), sex, and the pubertal stage reached.

The changes observed in the longitudinal data by the combined groups of obesity degree and presence of IR are shown in Table 4. Children with OW/OB who developed IR during puberty (group 5) presented a significant reduction in the concentration of 25(OH)D (Δ = −7.0 ng/mL; *p* = 0.035). Moreover, decreases in HDL-c (*p* = 0.020) and QUICKI (*p* < 0.001) and increases in insulin levels (*p* < 0.001), HOMA-IR (*p* < 0.001), TAG levels (*p* < 0.001), WC (*p* < 0.001), and systolic (*p* = 0.002) and diastolic (*p* = 0.005) blood pressure were also observed in this group. In addition, a significant correlation was found between 25(OH)D levels and TAG (r = −0.646, *p* = 0.017) and WC (r = −0.622, *p* = 0.023) in the pubertal stage in this group (data not shown).

Finally, negative associations were shown between the change over time (T1–T0) in TAG and WC with change over time (T1–T0) in 25(OH)D levels (B = −0.098, *p* = 0.034; B = −0.206, *p* = 0.035, respectively) after adjusting for the change in BMI z-score, sex, and the pubertal stage reached (Supplementary Table S2).

Associations between 25(OH)D levels and WC and TAG in each measurement time and delta values by obesity degree are shown in Supplementary Figures S2 and S3, respectively.

## 4. Discussion

Our findings showed that, in the pubertal period, the increase in insulin levels, HOMA-IR, TAG, and decrease in QUICKI was significantly associated with the reduction in 25(OH)D levels independently of sex, body mass, and the pubertal stage reached. However, no associations were found in the prepubertal stage when adjusting by the potential covariables. Additionally, non-insulin-resistant children with overweight or obesity that changed to insulin-resistant during puberty showed a significant decrease in the concentration of 25(OH)D over time accompanied by a reduction in HDL concentrations and an increase in the TAG levels and WC.

### 4.1. Deficiency of Vitamin D

Most of the worldwide population has vitamin D deficiency, which has been related, in part, to obesity [29]. In our pubertal sample, 92.6% of children with overweight and obesity showed non-adequate values of vitamin D (31.5% insufficiency and 61.1% deficiency), while out of the normal-weight children, 90.9% showed non-adequate values (27.3% insufficiency and 63.6% deficiency). Out of those that showed deficiency in the total pubertal sample, 70.2% had overweight or obesity and 29.8% were normal weight. Some mechanisms have been hypothesized to explain the relation between vitamin D deficiency and obesity, such as lower dietary intake of vitamin D, reduced skin exposure to sunlight due to limited time spent on outdoor physical activity [30], and 25(OH)D accumulation in fat and dilution volumetric of this vitamin due to a large amount of fat mass [6]. In this sense, all children evaluated in this study lived in a region above the 43rd parallel north, where they are considered at risk of vitamin D deficiency due to low sun exposure, especially during winter.

### 4.2. Insulin Resistance and Vitamin D

Previous studies have shown that vitamin D deficiency is related to IR and to alterations in the markers that characterize metabolically unhealthy individuals with obesity. In these studies, it has also been found that vitamin D deficiency increases the risk of cardiovascular disease in children with obesity [3,31,32,33]. However, there is a lack of studies showing that IR and the other cardiometabolic markers are predictors of vitamin D concentration. In our sample, we have found that, regarding IR and vitamin D, insulin levels and HOMA-IR were inversely associated with 25(OH)D concentration in children. Further, corroborating these relationships, insulin sensitivity (QUICKI index) was directly associated with concentrations of 25(OH).

The role of vitamin D status on glucose homeostasis can be explained by the fact that the active form of vitamin D (1,25[OH]_2_D) activates the transcription of the insulin receptor and peroxisome proliferator-activated receptor-δ genes and increases insulin-mediated glucose transport [34]. Moreover, this vitamin has been found to participate in the synthesis and improvement of insulin sensitivity due to the vitamin D receptor and enzyme 1-α-hydroxylase in pancreatic beta cells, the vitamin D response element in the promoter insulin gene, and the vitamin D receptor in skeletal muscle [34]. In addition, vitamin D has been linked to mitochondrial function in experimental studies [35,36], and the dysfunction of these organelles precedes the development of IR.

### 4.3. Puberty, Insulin Resistance, and Vitamin D

Previous longitudinal studies have suggested that puberty influences vitamin D status and its relation with IR and other metabolic syndrome components in the pediatric population [17,37,38]. In this sense, it has been shown that the prevalence of vitamin D deficiency increases among older children [39,40], and puberty seems to influence this deficiency [4,41], probably due to the increased requirement of vitamin D for growth and bone turnover [14]. It is worth noting that puberty is marked by an increase in body weight, which is associated with IR and a reduction in serum 25(OH)D levels [17,41,42,43]. However, previous studies found no associations between serum 25(OH)D and insulin parameters in prepubertal children [38,42]. This is in line with the cross-sectional results of the present study, where no significant associations were observed between HOMA-IR and the 25(OH)D levels in the prepubertal children. However, we did find cross-sectional associations between HOMA-IR and 25(OH)D levels in the pubertal period, suggesting that these associations could be triggered by puberty.

### 4.4. Obesity during Puberty, Insulin Resistance, and Vitamin D

We found that the development of IR during puberty in children with OW/OB was accompanied by a reduction in 25(OH)D and HDL-c levels and an increase in the concentrations of TAG. These outcomes increase the cardiovascular risk in this population.

Several studies have associated the development of obesity with puberty; however, this relationship has been shown to be bidirectional, i.e., excess weight can also influence puberty [44,45,46]. Proposed mechanisms for these relationships include the action of aromatase in adipose tissue, an enzyme that acts in the conversion of androgens to estrogens; the latter being known to increase body fat, favoring body weight gain [47,48]. Besides these, a direct action of adipokines has also been postulated, especially leptin, for its action in regulating the hypothalamic–pituitary–gonadal axis, signaling the release of gonadotropin-releasing hormone (GnRH), a mechanism necessary for the onset of puberty [48,49]. Moreover, worsening glucose tolerance associated with obesity is also another aspect observed in pubertal children compared to those in the prepubertal stage [50,51], which, added to the increased production of adipokines in obesity, increases the chances of developing IR during puberty [52].

It is already known that obesity is the main contributing factor to IR in children since the hypertrophy of adipose tissue in obese children associated with stromal hyperproliferation along with macrophage infiltration evidence the participation of inflammation in IR [53]. In addition to the previous explanation, obesity and IR are associated with the presence of chronic low-grade inflammation, situations that may benefit from adequate vitamin D status, given its action in reducing cytokine and chemokine release by adipocytes and chemotaxis of monocytes [31,54].

In a preclinical study, vitamin D insufficiency exacerbated macrophage recruitment, and adipose tissue increased in animals under high-fat diet conditions with a concomitant release of proinflammatory adipokines [54]. Several studies have already shown an inverse relationship between overweight or obesity and vitamin D deficiency in children and adolescents [4,55,56]. This relationship also was observed in a meta-analysis of epidemiological studies with adults, in which increased vitamin D concentrations reduced the risk of abdominal obesity in a dose-dependent manner [57]. Thus, adequate vitamin D levels may be even more necessary in the population with obesity than in the normal-weight population.

The relationship between obesity, IR, and vitamin D deficiency in the pediatric population is still not clarified, especially during puberty. Previous studies demonstrated that the serum 25(OH)D and IR in children with obesity are influenced by puberty [42,58]. In addition, another study showed that children with central obesity and suboptimal vitamin D levels before puberty onset had higher IR in the pubertal stage when compared with those without central obesity and optimal vitamin D levels [17]. These outcomes increase the cardiovascular risk in our population. In the present study, we have shown that cardiometabolic variables predict lower 25(OH)D levels at the pubertal stage. Additionally, associations between the increased TAG levels and reduction of 25(OH)D serum concentrations were found during puberty. Although studies that show associations in this direction are not found in the literature, vitamin D and dyslipidemias have been the focus of studies involving children with obesity [56,59] and in the pubertal stages [38,56].

### 4.5. Strengths and Limitations

Some limitations need to be considered for the present study. First, the small sample size; however, the lack of longitudinal studies should be taken into account. In addition, the study did not include data on diet or sun exposure. Considering that the children live in a region above the 43rd parallel north, a deficit risk zone for hours of sunlight and radiation angle, blood was collected in all seasons. On the other hand, this study presents the strength of having measured 25(OH)D levels and cardiometabolic risk markers at different stages of sexual maturation so that the influence of puberty on the outcomes of interest and the longitudinal associations could be assessed.

## 5. Conclusions

In conclusion, there is an association in puberty between some cardiometabolic factors and 25(OH)D levels, but this was not found in the prepubertal stage. Interestingly, non-insulin-resistant children with overweight or obesity that became insulin-resistant during puberty showed a significant decrease in the concentration of 25(OH)D over time and HDL-c while they showed a significant increase in WC and TAG levels. Additionally, the changes during puberty of the cardiometabolic factors, specifically TAG levels and WC, were negatively associated with changes in 25(OH)D levels, independently of body mass index. In addition, considering the endemic deficiency/insufficiency of vitamin D in the population, children with IR and obesity could present even lower levels of 25(OH)D along with other cardiometabolic disorders. These results highlight the importance of screening and preventing vitamin D deficiency during puberty to avoid cardiometabolic risk early in life and the utility of vitamin D as a cardiometabolic risk marker. Further longitudinal studies are needed to investigate those associations in depth.

## Figures and Tables

**Table 1:** Characteristics of the participants at baseline (prepubertal) and at the follow-up (pubertal) according to vitamin D status.

	T0 (Prepubertal)					T1 (Pubertal)					*p*-Value *
	All (*n* = 76)	Optimal (*n* = 14)	Insufficiency (*n* = 26)	Deficiency (*n* = 36)	*p*-Value	All (*n* = 76)	Optimal (*n* = 6)	Insufficiency (*n* = 23)	Deficiency (*n* = 47)	*p*-Value	
Age (years)	7.8 (1.9)	6.3 (1.6)	8.0 (1.9)	8.2 (1.7)	0.002	13.9 (2.2)	15.2 (1.6)	14.2 (2.4)	13.7 (2.2)	0.229	<0.001
Weight (kg)	37.9 (12.1)	32.0 (9.3)	38.4 (15.0)	39.9 (10.1)	0.07	68.0 (21.2)	72.7 (16.9)	69.1 (13.5)	66.8 (24.6)	0.619	<0.001
BMI (kg/m^2^)	22.8 [14.3–36.0]	22.8 [14.8–26.8]	22.4 [14.7–36.0]	22.9 [14.3–35.5]	0.519	26.2 [15.0–45.5]	25.9 [18.8–37.6]	25.9 [17.9–32.6]	26.5 [15.0–45.5]	0.997	<0.001 ^¥^
BMI z-score	2.1 (1.8)	2.4 (2.4)	2.0 (1.8)	2.1 (1.6)	0.785	1.7 (1.6)	1.5 (2.0)	1.5 (1.2)	1.8 (1.7)	0.799	0.003
Waist circumference (cm)	74.4 (13.1)	67.8 (11.2)	75.5 (15.1)	76.3 (11.9)	0.107	87.1 (15.5)	90.5 (19.9)	86.6 (11.7)	86.9 (16.7)	0.850	<0.001
SBP (mmHg)	104.3 (11.8)	100.1 (15.4)	104.9 (12.2)	105.5 (9.6)	0.333	116.2 (15.8)	118.9 (9.9)	114.6 (11.5)	115.7 (18.6)	0.841	<0.001
DBP (mmHg)	62.0 [45.0–100.0]	59.5 [46.0–79.0]	62.5 [49.0–77.0]	63.0 [45.0–100.0]	0.671	67.0 [49.5–94.5]	72.7 [63.0–82.0]	68.0 [55.0–85.0]	65.5 [49.5–94.5]	0.266	<0.001 ^¥^
25(OH)D (ng/mL)	23.0 (10.6)	40.9 (8.6)	24.4 (2.6)	15.1 (3.5)	<0.001	19.0 (7.6)	35.7 (6.9)	24.4 (2.5)	14.3 (3.6)	<0.001	0.004
Fasting glucose (mg/dL)	81.0 (8.2)	83.6 (6.7)	80.6 (8.4)	80.3 (8.6)	0.415	81.2 (7.4)	80.5 (5.0)	80.7 (7.7)	81.6 (7.6)	0.872	0.867
Fasting insulin (mUI/L)	8.0 (6.0)	6.4 (4.6)	7.4 (5.4)	9.1 (6.9)	0.245	14.2 (9.4)	15.9 (14.1)	11.4 (7.6)	15.4 (9.5)	0.123	<0.001
HOMA-IR	1.6 (1.2)	1.3 (0.9)	1.5 (1.1)	1.8 (1.4)	0.327	2.9 (2.0)	3.1 (1.9)	2.4 (2.0)	3.2 (3.0)	0.131	<0.001
QUICKI	0.4 (0.05)	0.4 (0.05)	0.4 (0.05)	0.4 (0.04)	0.409	0.3 (0.03)	0.3 (0.05)	0.3 (0.03)	0.3 (0.03)	0.115	<0.001
TAG (mg/dL)	56.2 (27.0)	48.1 (20.0)	51.7 (20.9)	62.6 (32.0)	0.211	70.7 (31.7)	56.8 (20.6)	64.1 (20.2)	75.7 (36.4)	0.222	<0.001
Cholesterol (mg/dL)	164.0 [102.0–298.0]	168.0 [112.0–221.0]	168.0 [102.0–225.0]	162.5 [104.0–298.0]	0.849	157.0 [101.0–271.0]	157.5 [126.0–200.0]	152.0 [101.0–210.0]	157.0 [103.0–271.0]	0.382	0.053 ^¥^
HDL-c (mg/dL)	52.6 (12.6)	53.3 (9.8)	52.1 (11.3)	52.7 (14.6)	0.958	50.6 (15.1)	49.5 (13.3)	45.5 (9.6)	53.2 (17.0)	0.163	0.180
LDL-c (mg/dL)	92.0 [52.0–224.0]	101.0 [52.0–155.0]	99.0 [56.0–139.0]	92.0 [56.0–224.0]	0.822	87.0 [50.0–187.0]	82.5 [71.0–113.0]	87.0 [50.6–147.0]	90.0 [50.0–187.0]	0.875	0.026 ^¥^
Adiponectin (mg/L)	17.9 (12.0)	24.9 (14.4)	16.9 (11.6)	14.6 (9.8)	0.036	12.0 (8.5)	12.5 (9.5)	9.6 (5.3)	13.2 (9.5)	0.582	0.001
Leptin (μg/L)	13.8 (13.4)	10.1 (7.9)	15.2 (17.1)	14.3 (12.1)	0.515	10.4 (7.5)	10.7 (12.2)	8.3 (6.2)	11.3 (7.4)	0.322	0.047

Data are expressed as mean (standard deviation) or median [min-max]. * Statistical differences between prepubertal stage and pubertal stage (all) (paired *t*-test or ^¥^ Wilcoxon test). *p <* 0.05 was considered significant. Abbreviations: BMI: Body Mass Index; DBP: Diastolic Blood Pressure; SBP: Systolic Blood Pressure; 25(OH)D: 25-hydroxycholecalciferol; HOMA-IR: Homeostasis Model Assessment for Insulin Resistance; QUICKI: Quantitative Insulin Sensitivity Check Index; TAG: Triglycerides; HDL-c: High-Density Lipoprotein Cholesterol; LDL-c: Low-Density Lipoprotein Cholesterol.

**Table 2:** Multivariable linear regression analysis between cardiometabolic variables and levels of 25(OH)D, both at the pubertal stage (T1).

Cardiometabolic Variables (T1)	25(OH)D Levels (T1) (ng/mL)					
	Unadjusted Model ^1^			Adjusted Model ^2^		
	B	95% CI	*p*-Value	B	95% CI	*p*-Value
Waist circumference (cm)	−0.007	−0.013 to −0.001	0.021	−0.002	−0.016 to 0.012	0.759
SBP (mmHg)	−0.006	−0.012 to 0.000	0.065	−0.002	−0.010 to 0.006	0.580
DBP (mmHg)	−0.059	−0.249 to 0.130	0.535	0.082	−0.138 to 0.302	0.459
Glucose (mg/dL)	−0.001	−0.014 to 0.012	0.859	−0.003	−0.016 to 0.010	0.652
Insulin (mUI/L)	−0.283	−0.437 to −0.128	0.001	−0.250	−0.446 to −0.054	0.013
HOMA-IR	−0.256	−0.404 to −0.108	0.001	−0.219	−0.400 to −0.038	0.019
QUICKI	1.817	0.795 to 2.839	0.001	1.574	0.337 to 2.811	0.013
TAG (mg/dL)	−0.349	−0.563 to −0.135	0.002	−0.274	−0.525 to −0.024	0.032
Cholesterol (mg/dL)	−0.018	−0.098 to 0.061	0.653	−0.041	−0.121 to 0.038	0.303
HDL-c (mg/dL)	−0.015	−0.107 to 0.077	0.748	−0.098	−0.201 to 0.005	0.061
LDL-c (mg/dL)	−0.011	−0.075 to 0.053	0.727	−0.019	−0.083 to 0.044	0.551
Adiponectin (mg/L)	0.062	−0.071 to 0.196	0.356	0.003	−0.155 to 0.161	0.973
Leptin (μg/L)	−0.080	−0.153 to −0.007	0.032	−0.014	−0.160 to 0.132	0.850

Multivariable linear regression analysis with cardiometabolic variables as independent variables in pubertal children (T1) ^1^. ^2^ The model was adjusted for sex and the BMI z-score, and the pubertal stage (T1). Abbreviations: BMI: Body Mass Index; HOMA-IR: Homeostasis Model Assessment for Insulin Resistance; QUICKI: Quantitative Insulin Sensitivity Check Index; DBP: Diastolic Blood Pressure; SBP: Systolic Blood Pressure; TAG: Triacylglycerol; HDL-c: High-Density Lipoprotein Cholesterol; LDL-c: Low-Density Lipoprotein Cholesterol; SE: Standard Error; CI: Confidence Interval.

**Table 3:** Multivariable linear regression analysis between cardiometabolic variables at the prepubertal stage (T0) and 25(OH)D levels in the pubertal stage (T1).

Cardiometabolic Variables (T0)	25(OH)D Levels (T1) (ng/mL)					
	Unadjusted Model ^1^			Adjusted Model ^2^		
	B	95% CI	*p*-Value	B	95% CI	*p*-Value
Waist circumference (cm)	−0.004	−0.011 to 0.003	0.272	0.004	−0.005 to 0.013	0.398
SBP (mmHg)	−0.007	−0.015 to 0.002	0.110	−0.004	−0.012 to 0.005	0.394
DBP (mmHg)	−0.136	−0.295 to 0.023	0.093	−0.076	−0.238 to 0.086	0.352
Glucose (mg/dL)	−0.001	−0.012 to 0.011	0.875	0.002	−0.011 to 0.014	0.774
Insulin (mUI/L)	−0.136	−0.258 to −0.014	0.030	−0.097	−0.228 to 0.034	0.144
HOMA-IR	−0.132	−0.252 to −0.012	0.032	−0.088	−0.214 to 0.039	0.172
QUICKI	0.783	0.025 to 1.541	0.043	0.534	−0.254 to 1.321	0.181
TAG (mg/dL)	−0.137	−0.344 to 0.071	0.193	−0.007	−0.227 to 0.214	0.953
Cholesterol (mg/dL)	−0.008	−0.080 to 0.065	0.836	−0.004	−0.078 to 0.071	0.917
HDL-c (mg/dL)	−0.006	−0.112 to 0.100	0.910	−0.077	−0.184 to 0.030	0.157
LDL-c (mg/dL)	−0.004	−0.067 to 0.058	0.888	0.003	−0.060 to 0.065	0.935
Adiponectin (mg/L)	0.140	0.000 to 0.279	0.049	0.070	−0.085 to 0.225	0.373
Leptin (μg/L)	−0.006	−0.061 to 0.049	0.835	0.046	−0.015 to 0.108	0.136

Multivariable linear regression analysis with cardiometabolic variables as independent variables in prepubertal children (T0) ^1^. ^2^ The adjusted model was adjusted for 25(OH)D levels in prepubertal stage (T0), BMI z-score (T1), sex, and the pubertal stage reached ^2^. Abbreviations: BMI: Body Mass Index; HOMA-IR: Homeostasis Model Assessment for Insulin Resistance; QUICKI: Quantitative Insulin Sensitivity Check Index; DBP: Diastolic Blood Pressure; SBP: Systolic Blood Pressure; TAG: Triacylglycerol; HDL-c: High-Density Lipoprotein Cholesterol; LDL-c: Low-Density Lipoprotein Cholesterol; SE: Standard Error; CI: Confidence Interval.

**Table 4:** Characteristics of the participants of the longitudinal study according to obesity degree and presence of IR.

Variables	NW Non-IR No Change Group 1 (*n* = 16)		OW/OB Non-IR to NW Non-IR Group 2 (*n* = 6)		OW/OB Non-IR No Change Group 3 (*n* = 26)		OW/OB—IR to Non-IR Group 4 (*n* = 9)		OW/OB—Non-IR to IR Group 5 (*n* = 13)		OW/OB IR No Change Group 6 (*n* = 6)	
	T0	∆	T0	∆	T0	∆	T0	∆	T0	∆	T0	∆
Sex, F/M	4/12		4/2		8/18		6/3		8/5		4/2	
Age (years)	7.5 (2.1)	7.3 (3.2) **	8.4 (1.6)	6.4 (2.5) **	7.8 (1.9)	5.9 (2.5) **	8.1 (1.9)	5.1 (3.1) **	7.1 (1.9)	6.5 (2.8) **	8.6 (0.8)	4.9 (1.9) **
BMI z-score	−0.3 (0.6)	0.1 (0.5)	1.3 (0.6)	−1.4 (0.4) **	3.0 (1.3)	−1.0 (1.5) **	3.1 (1.9)	−1.0 (1.7)	2.5 (1.1)	0.4 (1.3)	3.5 (1.6)	0.4 (0.9)
BMI (kg/m^2^) ^¥^	15.9 [14.3; 20.2]	3.4 [0.5; 8.7] **	21.6 [17.7; 24.1]	−0.6 [−3.9; 2.8]	23.6 [19.6; 29.8]	3.07 [−3.9; 10.7] **	25.4 [21.4; 36.0]	0.8 [−5.2; 11.3]	23.3 [17.9; 25.1]	5.1 [2.5; 15.6] **	26.2 [23.0; 35.5]	7.1 [2.0; 12.5] *
Waist circumference (cm)	58.3 (6.1)	13.2 (7.2) **	75.7 (9.4)	−0.2 (9.9)	78.0 (10.2)	10.8 (11.7) **	81.6 (13.1)	10.2 (16.3)	75.4 (8.5)	18.9 (11.1) **	89.5 (12.4)	22.4 (13.6) *
SBP (mmHg)	100.7 (11.9)	7.2 (15.2)	105.0 (10.6)	7.8 (17.9)	103.8 (13.5)	12.5 (16.1) **	109.2 (13.6)	11.8 (15.0)	102.5 (9.0)	13.8 (12.8) **	111.5 (7.3)	24.1 (29.6)
DBP (mmHg)	59.5 [50.0–72.0]	6.0 [−13.5; 20.5]	68.5 [58.0; 76.0]	−6.0 [−18.0; 3.0]	62.0 [46.0; 81.0]	7.0 [−11.0; 21.0] **	66.0 [50; 79]	0.5 [−11.0; 31.5]	63.0 [45; 71.0]	9.0 [−4.0; 24.0] **	64.5 [56.0; 100.0]	7.7 [−20.0; 21.5]
25(OH)D (ng/mL)	27.1 (15.2)	−7.6 (13.4)	21.5 (6.8)	−0.5 (12.0)	22.2 (8.1)	−1.2 (10.8)	24.4 (9.4)	−6.3 (9.4)	23.0 (10.8)	−7.0 (12.4) *	15.0 (6.3)	0.1 (10.3)
Fasting glucose (mg/dL)	80.0 (9.2)	0.6 (10.5)	83.3 (8.1)	−3.0 (9.0)	80.4 (7.9)	−0.5 (10.2)	81.1 (7.9)	−3.3 (7.8)	79.4 (6.9)	5.5 (11.8)	87.5 (9.9)	−0.5 (17.8)
Fasting insulin (mUI/L)	4.7 (2.8)	5.3 (3.3) **	5.6 (3.3)	2.7 (3.1)	5.3 (3.2)	4.3 (4.7) **	17.0 (6.3)	−4.2 (7.2)	7.8 (3.2)	17.3 (8.0) **	18.1 (5.3)	11.9 (11.7) *
HOMA-IR	0.9 (0.5)	1.1 (0.7) **	1.1 (0.6)	0.5 (0.7)	1.1 (0.7)	0.8 (0.9) **	3.3 (0.9)	−0.9 (1.4)	1.5 (0.7)	3.8 (2.2) **	3.9 (1.1)	2.3 (1.8) **
QUICKI	0.4 (0.04)	−0.05 (0.04) **	0.4 (0.04)	−0.03 (0.04)	0.4 (0.04)	−0.05 (0.05) **	0.3 (0.01)	0.02 (0.03)	0.4 (0.03)	−0.06 (0.03) **	0.3 (0.01)	−0.02 (0.01) **
TAG (mg/dL)	44.8 (19.9)	12.8 (20.5) *	57.7 (24.4)	−3.0 (14.1)	52.7 (21.3)	13.6 (26.0) **	71.7 (26.5)	−10.1 (27.1)	55.7 (36.1)	43.8 (31.0) **	77.8 (34.3)	13.5 (36.6)
Cholesterol (mg/dL) ^¥^	175.0 [112.0–231.0]	−5.0 [−28.0; 43.0]	160.0 [119.0; 246.0]	9.5 [−36.0; 25.0]	173.0 [104.0; 298.0]	−7.0 [−101.0; 30.0]	164.0 [141.0; 221.0]	−14.0 [–39.0; 0.0] ^*^	158.0 [102.0; 185.0]	7.0 [−30.0; 33.0]	171.0 [135.0; 203. 0]	−11.5 [−36.0; 29.0]
LDL-c (mg/dL) ^¥^	102.0 [54.0–140.0]	−1.5 [−32.0; 25.0]	99.5 [56.0; 184.0]	3.0 [−30.0; 9.0]	101.0 [56.0; 224.0]	−5.0 [−81.0; 24.0]	83.0 [66.0; 155.0]	−5.0 [−35.0; 10.0]	92.0 [52.0; 114.0]	2.0 [−31.0; 28.0]	96.0 [62.0; 149.0]	−8.0 [−29.0; 15.0]
HDL-c (mg/dL)	59.7 (13.4)	0.2 (11.6)	51.3 (7.9)	6.7 (10.6)	53.7 (12.9)	−6.0 (15.1)	51.0 (11.9)	2.3 (28.9)	46.0 (9.8)	−3.7 (5.0) *	46.8 (12.8)	−2.3 (12.0)
Adiponectin (mg/L)	23.3 (14.6)	−9.0 (15.4) *	17.4 (11.4)	0.1 (15.6)	17.6 (13.1)	−6.5 (15.1) *	15.3 (10.0)	−1.9 (11.4)	11.4 (4.3)	−3.6 (6.2)	18.2 (11.6)	−0.8 (8.8)
Leptin (μg/L)	3.7 (6.2)	−0.1 (5.8)	9.4 (5.1)	−5.5 (4.22) *	14.2 (11.3)	−4.2 (12.3)	22.2 (22.0)	−11.4 (21.6)	13.5 (4.2)	3.0 (6.2)	34.5 (9.3)	−11.2 (11.2)

Data are expressed as mean (standard deviation) or median [min; max]. * *p* < 0.05; ** *p* < 0.01—statistical differences between prepubertal stage and pubertal stage (all) (paired *t*-test or ^¥^ Wilcoxon test). Δ: changes (T1–T0). Abbreviations: NW: Normal Weight; OW: Overweight; OB: Obesity; F: Female; M: Male; BMI: Body Mass Index; DBP: Diastolic Blood Pressure; SBP: Systolic Blood Pressure; 25(OH)D: 25-hydroxycholecalciferol; HOMA-IR: Homeostasis Model Assessment for Insulin Resistance; QUICKI: Quantitative Insulin Sensitivity Check Index; TAG: Triglycerides; HDL-c: High-Density Lipoprotein Cholesterol; LDL-c: Low-Density Lipoprotein Cholesterol.

## Supplementary Materials

The following are available online at https://www.mdpi.com/article/10.3390/nu13124488/s1, Supplementary Figure S1: Study design and experimental details of the study population, Supplementary Figure S2: Associations between 25(OH)D levels and WC in prepubertal (T0) and pubertal stages (T1), and delta values, by obesity degree, Supplementary Figure S3: Associations between 25(OH)D levels and TAG in prepubertal (T0) and pubertal stages (T1), and delta values, by obesity degree. Supplementary Table S1: Percentage of prepubertal and pubertal children according to vitamin D status and obesity degree. Supplementary Table S2: Multivariable regression analysis between change of cardiometabolic variables and change in 25(OH)D levels (delta values T1–T0).
