# Supplementary material for: The Vitamin D Decrease in Children with Obesity Is Associated with the Development of Insulin Resistance during Puberty: The PUBMEP Study
Source: Nutrients. 2021 Dec 15;13(12):4488. doi: 10.3390/nu13124488 (PMC8709093; doi:10.3390/nu13124488)
Supplement: Supplementary file 1 [file nutrients-13-04488-s001.zip › suplemental material/aguilera Supplementary Tables_16_11_21.pdf]

Supplementary Table S1. Percentage of prepubertal and pubertal children according to vitamin D status and obesity degree.

Variables	T0 (Prepubertal)				T1 (Pubertal)			
	Vitamin D status, n (%)				*Vitamin D status, n (%)*			
	Optimal	Insufficiency	Deficiency	*p*-value*	Optimal	Insufficiency	Deficiency	*p*-value*
Obesity degree, n (%)								
Normalweight	4 (25.0%)	7 (43.7%)	5 (31.3%)		2 (9.1%)	6 (27.3%)	14 (63.6%)	
Overweight	2 (11.1%)	6 (33.3%)	10 (55.6%)	0.616	3 (13.0%)	7 (30.5%)	13 (56.5%)	0.749
Obesity	8 (19.0%)	13 (31.0%)	21 (50%)		1 (3.2%)	10 (32.3%)	20 (64.5%)	

*Statistical differences between prepubertal stage and pubertal stage (chi-square test). $p<0.05$ was considered significant.

Supplementary Table S2. Multivariable regression analysis between change of cardiometabolic variables and change 25(OH)D levels (delta values T1-T0).

Change Cardiometabolic Variables (T1-T0)	Change 25(OH)D levels (T1-T0)					
	Unadjusted model[1]			Adjusted model[2]		
	B	95% CI	*P*-value	B	95% CI	*P*-value
**Waist circumference (cm)**	-0.201	-0.372 to -0.030	0.**022**	-0.206	-0.396 to -0.015	**0.035**
**SBP (mmHg)**	-0.150	-0.316 to 0.016	0.077	-0.115	-0.291 to 0.061	0.198
**DBP (mmHg)**	-0.090	-0.352 to 0.173	0.499	-0.066	-0.329 to 0.198	0.621
**Glucose (mg/dL)**	0.168	-0.076 to 0.412	0.173	0.048	-0.243 to 0.339	0.746
**Insulin (mU/L)**	-0.265	-0.645 to 0.115	0.169	-0.300	-0.702 to 0.102	0.141
**HOMA-IR**	-1.018	-2.903 to 0.867	0.285	-1.048	-3.053 to 0.958	0.301
**QUICKI**	14.265	-46.778 to 75.308	0.643	8.020	-56.788 to 72.829	0.806
**TAG (mg/dL)**	-0.084	-0.173 to 0.004	0.060	-0.098	-0.188 to -0.008	**0.034**
**Cholesterol (mg/dL)**	-0.019	-0.137 to 0.100	0.757	-0.057	-0.179 to 0.065	0.354
**HDL-c (mg/dL)**	0.007	-0.164 to 0.177	0.939	-0.016	-0.184 to 0.153	0.851
**LDL-c (mg/dL)**	-0.005	-0.145 to 0.134	0.941	-0.048	-0.189 to 0.094	0.503
**Adiponectin (mg/L)**	0.306	0.089 to 0.523	**0.006**	0.232	-0.033 to 0.497	0.085
**Leptin (µg/L)**	0.033	-0.324 to 0.389	0.856	-0.182	-0.613 to 0.249	0.401

Multivariable regression analysis with change of cardiometabolic variables (T1-T0) as independent variable[1]. The adjusted model was adjusted for change of BMI z-score, sex, and the pubertal stage reached[2]. Abbreviations: BMI: Body Mass Index; HOMA-IR: Homeostasis Model Assessment for Insulin Resistance; QUICKI: Quantitative Insulin Sensitivity Check Index; DBP: Diastolic Blood Pressure; SBP: Systolic Blood Pressure; TAG: Triacylglycerols; HDL-c: High-Density Lipoproteins-cholesterol; LDL-c: Low-Density Lipoproteins-cholesterol; SE: Standard Error; CI: confidence interval.
