# Supplementary material for: The Vitamin D Decrease in Children with Obesity Is Associated with the Development of Insulin Resistance during Puberty: The PUBMEP Study
Source: Nutrients. 2021 Dec 15;13(12):4488. doi: 10.3390/nu13124488 (PMC8709093; doi:10.3390/nu13124488)
Supplement: Supplementary file 1 [file nutrients-13-04488-s001.zip › suplemental material/Sup_FigureS1_Nutrients.pdf]

Supplementary Figure S1. Study design and experimental details of the study population.

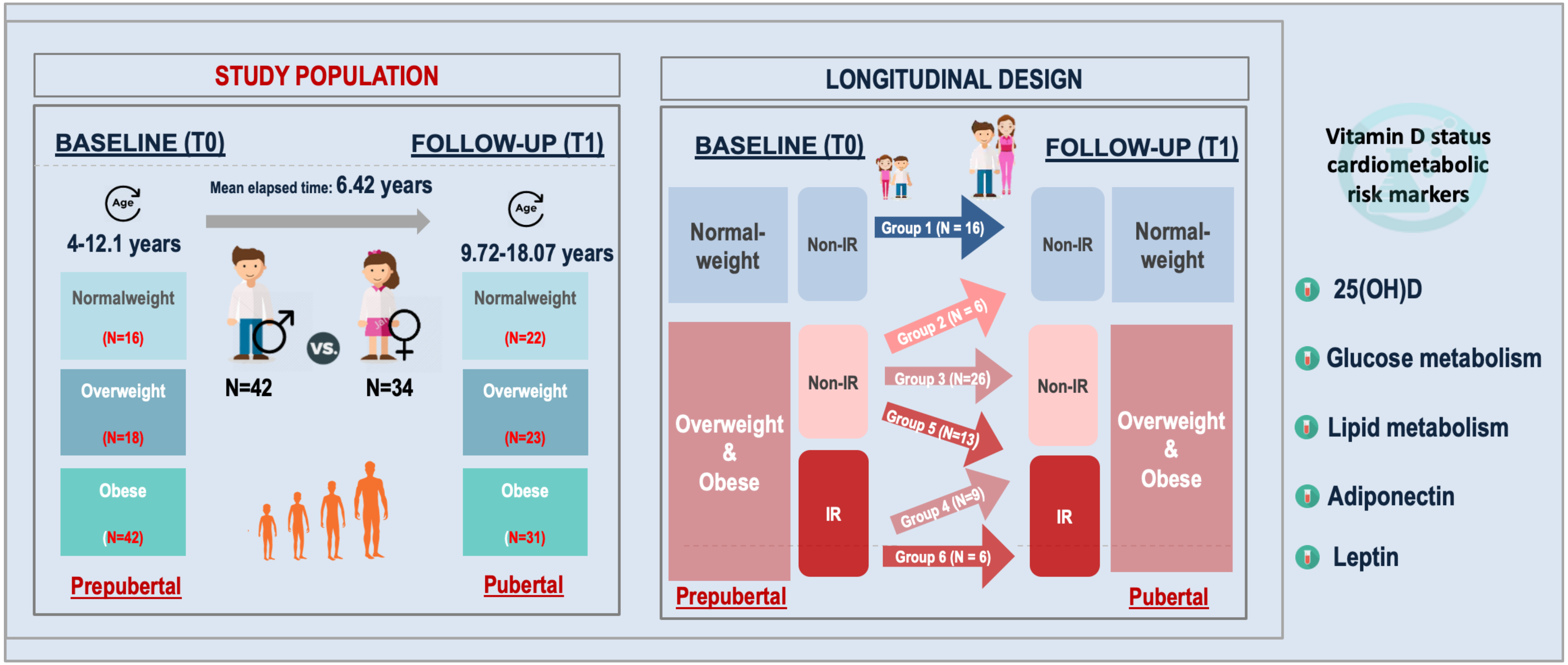

Abbreviations: IR: insulin resistance; 25(OH)D: 25-hydroxycholecalciferol.
