# Supplementary figures and images for: The Vitamin D Decrease in Children with Obesity Is Associated with the Development of Insulin Resistance during Puberty: The PUBMEP Study
Source: Nutrients. 2021 Dec 15;13(12):4488. doi: 10.3390/nu13124488 (PMC8709093; doi:10.3390/nu13124488)

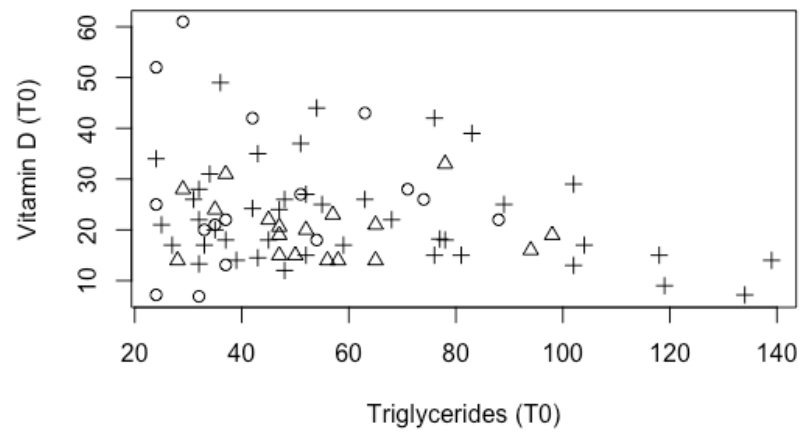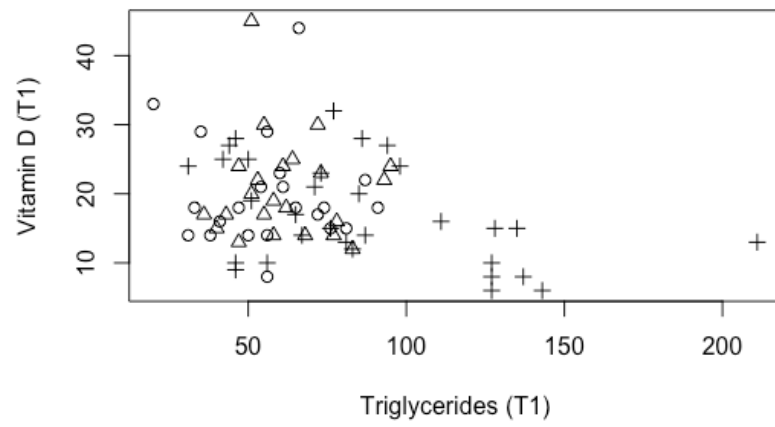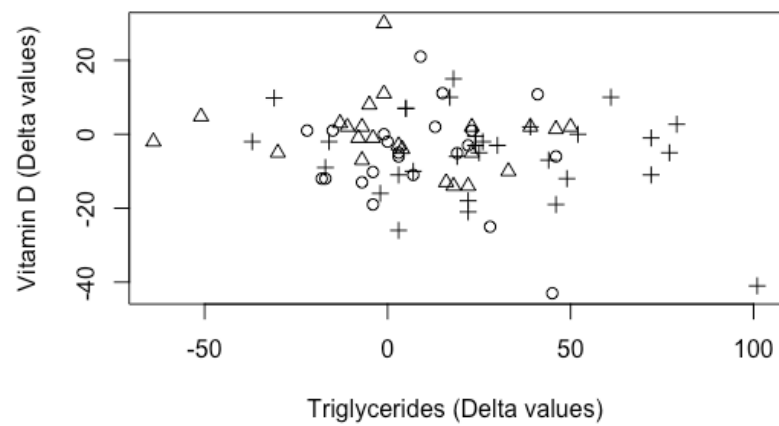

- + Children with obesity
- △ Children with overweight
- Children with normal weight

Supplement: Supplementary file 1 [file nutrients-13-04488-s001.zip › suplemental material/VITDandTG_SUP_FigS2.pdf]

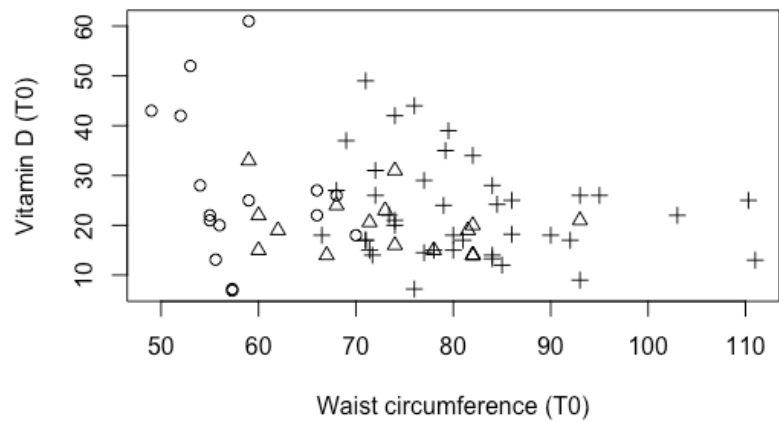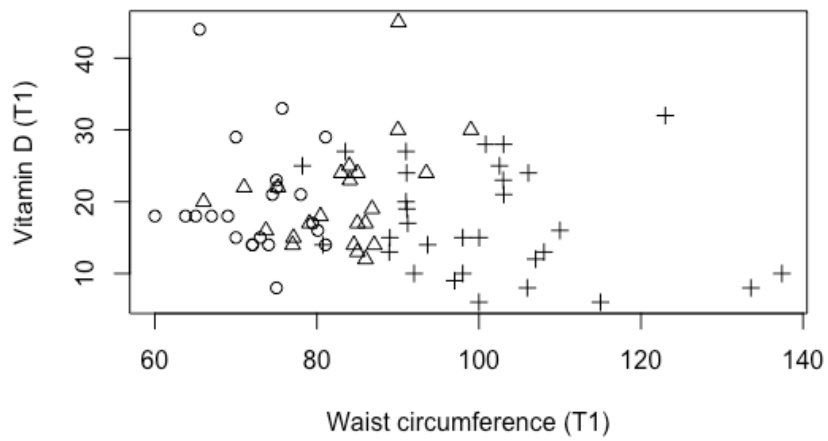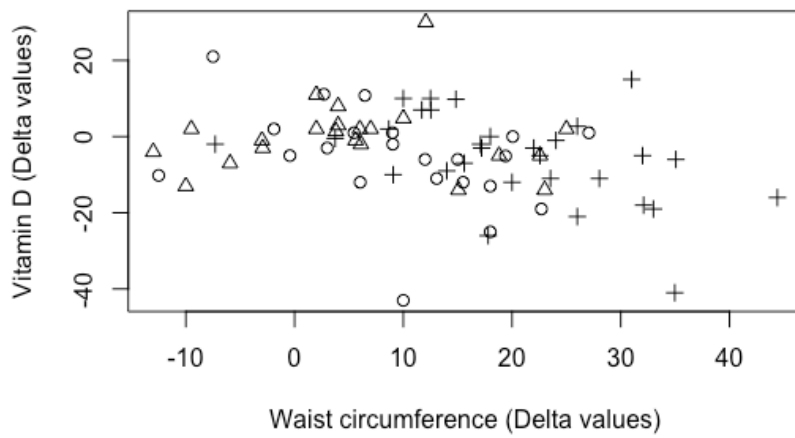

- + Children with obesity
- △ Children with overweight
- Children with normal weight

Supplement: Supplementary file 1 [file nutrients-13-04488-s001.zip › suplemental material/VITDandWC_SUP_FigS3.pdf]
